# Infectious diseases pharmacy practice, education, and research in Saudi Arabia: A review and future perspectives by the Infectious Diseases Pharmacy Specialty Network at the Saudi Society of Clinical Pharmacy

**DOI:** 10.1016/j.jsps.2022.10.014

**Published:** 2022-11-03

**Authors:** Yazed Saleh Alsowaida, Abrar K. Thabit, Thamer A. Almangour, Khalid Bin Saleh, Ahmad Mahrous, Masaad Saeed Almutairi, Bashayer Alshehail, Doaa Aljefri, Yahya Mohzari, Wafa Alfahad, Abdulah Almohaizeie, Khalid Eljaaly

**Affiliations:** a Department of Clinical Pharmacy, College of Pharmacy, Hail University, P.O. Box 6166, Hail 81442, Saudi Arabia; b Division of Infectious Diseases, the Warren Alpert Medical School, Brown University, Providence, RI 02903, USA; c Pharmacy Practice Department, Faculty of Pharmacy, King Abdulaziz University, Jeddah, Saudi Arabia; d Department of Clinical Pharmacy, College of Pharmacy, King Saud University, P.O. Box 2457, Riyadh 11451, Saudi Arabia; e College of Pharmacy, King Saud bin Abdulaziz University for Health Sciences, P.O. Box 3660, Riyadh 11481, Saudi Arabia; f King Abdullah International Medical Research Center, P.O. Box 3660, Riyadh 11481, Saudi Arabia; g Pharmaceutical Care Department, King Abdulaziz Medical City, P.O. Box 3660, Riyadh 11481, Saudi Arabia; h Department of Clinical Pharmacy, College of Pharmacy, Umm Al Qura University, Makkah, Saudi Arabia; i Department of Pharmacy Practice, College of Pharmacy, Qassim University, Qassim 51452, Saudi Arabia; j Pharmacy Practice Department, College of Clinical Pharmacy, Imam Abdulrahman Bin Faisal University, Dammam 31441, Saudi Arabia; k Department of Pharmacy, King Abdulaziz Medical City, Jeddah, Saudi Arabia; l Pharmaceutical Care Division, King Saud Medical City, Riyadh, Saudi Arabia; m Department of Pharmacy Services, Prince Sultan Military Medical City, Riyadh, Saudi Arabia; n Pharmaceutical care division, King Faisal Specialist Hospital and Research Center and, Faculty of Pharmacy and Medicine, Alfaisal University, Riyadh, Saudi Arabia

**Keywords:** Clinical pharmacy, Infectious disease, Pharmacist, Antimicrobial stewardship, Antibiotic stewardship, Saudi

## Abstract

**Background:**

Infectious diseases (ID) pharmacy is one of the rapidly evolving clinical pharmacy specialties in the Kingdom of Saudi Arabia (KSA). There are gaps in the literature on ID pharmacy status in KSA. This review aimed to provide an update on the current status of several areas related to ID pharmacy in KSA, including practice, education, and research, and make pertinent recommendations for future development to achieve the KSA Vision, 2030, KSA Vision, 2030.

**Methods:**

This review was developed by a group of ID pharmacists working in different sectors under the umbrella of the ID Pharmacy Specialty Network (PSN) of the Saudi Society of Clinical Pharmacy (SSCP). The authors evaluated domains related to ID pharmacy in KSA and searched the literature for relevant articles. Based on the experts' assessment of the current gaps and challenges, recommendations were made for future improvement.

**Results:**

Several aspects of ID pharmacy in KSA were evaluated, including history and development, antimicrobial resistance (AMR), antimicrobial stewardship programs (ASP), roles of ID pharmacists, ID pharmacy education, and research. The biggest challenges include AMR, the varying levels of ASP implementation, and the low number of ID-trained pharmacists, especially in non-major cities. Several recommendations for improvement were discussed.

**Conclusion:**

Infectious diseases pharmacy has sustained remarkable progress in KSA in several areas. However, more efforts are needed to increase ASP implementation, increase the number of ID-trained pharmacists, and encourage ID pharmacists in publishing and participating in practice guidelines, which will eventually help achieve the KSA Vision, 2030, KSA Vision, 2030.

## 1 Introduction

The KSA is a Middle Eastern country located in southwest Asia with a total estimated population of 33,413,660 people (of whom, 20,768,627 are Saudi citizens) as of 2021 (General Authority of Statistics, 2021). As of 2020, KSA has a Gross Domestic Product (GDP) of $793 billion and a GDP per capita of $48,948. Based on the United States (US) News & World Report, KSA is ranked 35 among the overall best countries (US News, Saudi Arabia, 2020). The 2000 World Health Organization (WHO) report for health systems ranked the healthcare system in KSA 26th out of 190 countries (WHO World Health Systems). Generally, KSA offers free access to all public healthcare services for its citizens through the Ministry of Health (MOH), which is funded by the KSA government (Al Asmri et al., n.d., Almalki et al., 2011). The healthcare system in KSA is comprised of the public (government-funded) healthcare sector and the private healthcare sector. In 2019, the governmental healthcare budget was $46 billion which represents 6.4 % of the GDP in 2018. The levels of the healthcare system in KSA are diversified and composed of primary care (basic curative and preventative care), secondary care (advanced acute care hospitals to provide diagnostics and surgical and rehabilitation services), and tertiary hospitals (complex cases that require referral to specialized hospitals) (Al Asmri et al., n.d., Almalki et al., 2011). Moreover, a cross-sectional study by AlRuthia et al. in 2018 on the statistics of the pharmacy workforce in KSA revealed that 18.5 % of the pharmacists were Saudi nationals and 85.1 % of them were males with 41.4 % of the pharmacy workforce in Riyadh region (AlRuthia et al., 2018). Notably, among the Saudi nationals in the study, there were only 66 (1.46 %) clinical pharmacists.

In 2016, KSA introduced a national transformational program (KSA Vision, 2030, KSA Vision, 2030) that has several national development initiatives. Regarding the healthcare sector transformation, the KSA Vision, 2030, KSA Vision, 2030 aims to facilitate access to healthcare services, improve quality and efficiency, improve health promotion and prevention services, and increase life expectancy (KSA Vision, 2030, KSA Vision, 2030, Healthcare). The pharmacy profession is an integral part of supporting KSA’s Vision 2030 (Badreldin et al., 2020). In 2014, another important milestone for healthcare is that KSA joined the World Health Security Agenda that aims to create global collaboration to increase global safety from ID, specifically relating to biosafety and biosecurity (MOH Annual Report).

Antimicrobial resistance (AMR) is a global healthcare problem that is associated with adverse health consequences and economic burdens (Thabit et al., 2015). In the KSA, there is an increasing prevalence of extended-spectrum beta-lactamase (ESBL) producing bacteria, where some institutions had 29 % ESBL rates among *Escherichia coli* and 65 % ESBL rates among *Klebsiella pneumoniae* (Zowawi et al., 2015). In addition, Gram-negative bacilli have shown a substantial increase in the rate of carbapenem resistance over the last decade compared with the 1990 s. Al Salman et al. evaluated Gram-Negative infections for critical priority pathogens per the WHO and found that KSA has a prevalence of 80 % for carbapenem-resistant *Acinetobacter baumannii,* 40 % for ESBL, 30 % for carbapenem-resistant *Pseudomonas aeruginosa,* and 15 % for carbapenem-resistant *Enterobacteriaceae* (Al Salman et al., 2020). A study conducted in the Medina region in KSA evaluating the resistance rate of *K. pneumoniae* reported resistance rates of 77 % to ceftriaxone and 46 % to carbapenems (Al-Zalabani et al., 2020). A recent multicenter study by Alraddadi et al. found that OXA-48 was the most prevalent carbapenem-resistant Enterobacterales in Saudi Arabia, and about a third of patients died within 30 days (Alraddadi et al., 2022). Moreover, several studies have reported increasing rates of multidrug-resistant *Psuedomonas aeruginosa* and *Acinetobacter baumannii* (Al-Obeid et al., 2015, Khan and Faiz, 2016). Major reasons for AMR are overprescribing antibiotics by healthcare workers, inappropriate use of broad-spectrum antibiotics, and inadequate implementation of antimicrobial stewardship programs (ASPs) in the KSA (Benmerzouga et al., 2019). In an effort to combat AMR, in 2018, KSA enacted legislation banning antibiotics sales in the community pharmacies without a prescription and imposing a 100,000 Saudi Riyal fine and revoking the pharmacy licensure (Al-Jedai et al., 2022). Enacting such a law is expected to improve antimicrobial use in community settings.

Clinical pharmacy has advanced remarkably in the past 3 decades in KSA in several fields that include ID. Clinical pharmacists specialized in ID play a major role in improving treatment outcomes and reducing AMR rates and healthcare costs. Currently, there is a gap in the literature on several aspects of the status of ID pharmacy in KSA. The objective of this review is to shed the light on several areas related to ID pharmacy, such as clinical practice and services, education, and research and propose recommendations to advance ID pharmacy to achieve the KSA Vision, 2030, KSA Vision, 2030.

## 2 Methods

The review was developed by a group of ID pharmacists working in hospital and academic settings in KSA under the umbrella of the ID PSN of the SSCP. The ID PSN of SCCP was established in 2020 and it is composed of a group of clinical pharmacists specialized in ID that focuses on advancing ID pharmacy in many areas, such as pharmacy practice and education. The authors identified the domains relevant to ID pharmacy in KSA to be included in the article. PubMed and Google Scholar databases were searched to identify published literature articles using the following keywords: “infectious diseases” AND “pharmacy” AND “KSA.” Some of the data reported in the article are based on expert opinions that lack published data. Then, based on the experts' assessment of the current gaps and challenges, the authors made recommendations for future improvement of ID pharmacy and to achieve the KSA Vision, 2030, KSA Vision, 2030.

## 3 History of clinical pharmacy in KSA

The pharmacy profession has advanced rapidly in KSA with the advancement of education, technology, and automation in all pharmacy operations in the healthcare system (Al-Jedai et al., 2016). The clinical pharmacy profession plays a major role in tailoring pharmacotherapeutic plans for patients; thus, providing safe and effective treatment while saving costs to the healthcare systems. Clinical pharmacy in KSA has been largely influenced by the US clinical pharmacy model and has remarkably evolved over the past 3 decades (Badreldin et al., 2020). Currently, there are more than 20 colleges of pharmacy across KSA that offer Doctor of Pharmacy (PharmD) degree programs. In fact, the vast majority of clinical pharmacy leaders and advocates in KSA have been trained in the US, where they have completed general and specialized clinical pharmacy residencies, and many have completed research fellowships (Badreldin et al., 2021).

In 1997, the first pharmacy residency program was established in KSA at King Faisal Specialist Hospital and Research Center in collaboration with St. Louis College of Pharmacy, MO, US. As of 2022, there are more than 160 general pharmacy residency positions in more than 25 hospitals in KSA. Notably, some of the residency programs in KSA are accredited by the American Society of Health-system Pharmacists (ASHP). Clinical pharmacy has a strong presence in several specialties. The infectious diseases pharmacy specialty is one of the cornerstones of pharmacy practice and education in the KSA (Badreldin et al., 2020). Pharmacy Residency programs in KSA include 2 years of general pharmacy practice (R1 and R2), which is equivalent to post-graduate year-1 (PGY-1) in the US, with the option of taking a third-year specialty (*R*3) in several areas including ID, which is equivalent to post-graduate year-2 (PGY-2) in the US (Al-Jedai et al., 2016).

## 4 Roles of ID pharmacists in KSA

Infectious diseases pharmacists play major roles as members of the multidisciplinary ID team and pharmacy experts’ members of the ASP or antimicrobial committee, and faculty members in the KSA universities. The ID pharmacist in KSA provides clinical, administrative, educational, and scholarly services. Such services benefit a wide variety of populations, including patients, physicians, medical trainees, pharmacists, pharmacy residents and students, as well as the public.

Infectious diseases pharmacists are an integral part of several healthcare and academic sectors and can provide numerous services. A detailed description of services provided by ID pharmacists in KSA is available in Table 1. Of note, this is a comprehensive list as some hospitals may have limited resources. However, ID pharmacists strive to make an impact that other healthcare workers recognize and appreciate. During COVID-19, for instance, ID pharmacists in KSA participated in emergency preparedness, were members of the COVID-19 medical team, participated in the development of the hospital’s COVID-19 management protocols, educated healthcare professionals and the public on the disease, and published relevant studies (Goff et al., 2020). Clinical pharmacy faculty members specialized in ID have also updated their didactic courses to include updated contents on COVID-19, which was also taught to pharmacy trainees of residents and interns. When the vaccine became available, immunization-certified ID pharmacists also participated in COVID-19 vaccine administration and training of vaccinators (Thabit and Jose 2022).

**Table 1:** Services provided by ID pharmacists in KSA.

**Discipline**	**Roles and Services**
Clinical	• Member of the multidisciplinary ID team, where they round on inpatients and assist with pharmacotherapeutic planning. Apply PK/PD principles in clinical settings to perform TDM of antimicrobials. Prospective audit and feedback of antimicrobials as they review patients receiving antimicrobials and evaluate appropriateness. Answering drug information questions pertinent to ID pharmacotherapy from the medical team and contacting pharmaceutical companies when needed. Providing outpatient services by working in ID clinics, including HIV and/or hepatitis clinics in collaboration with gastroenterology. Reviewing patients receiving IV antimicrobials for possible switching to PO therapy. Providing counseling on antimicrobial usage and monitoring for inpatients to be discharged. Manage OPAT service in hospitals in coordination with the nursing staff. Order/borrow antibiotics from other hospitals. Promote adherence to hospital policies and procedures. Encourage maintenance of routine immunization for patients and health staff members.
Administration	• Drug experts in ASP, where they develop antibiotic-based protocols, such as TDM and participate in developing hospital-specific guidelines for different ID and antimicrobials. Assist the Pharmacy & Therapeutics Committee on making formulary decisions on new antimicrobials. Medication use evaluation of antimicrobials to track compliance with ASP protocols and align results with susceptibility data of the hospital’s antibiogram. Develop antibiotics administration protocols and order set for common ID. Track ASP metrics, such as DOT and DDD by generating reports of antimicrobial use and analyzing them regularly. Assess antibiogram development.
Education and Training	• Training of clinical pharmacy residents (whether ID-specialized or other specialties requiring ID rotation) and students of Master of Science in clinical pharmacy. Training of pharmacy students in their advanced pharmacy practice experience year, as well as summer training. Participate in the weekly ID grand rounds by the ID team. Participate in hospital grand rounds. Teaching of medical ID fellows. Educate hospital nurses on variety of topics related to ID pharmacy; for example, prolonged infusion of β-lactam antibiotics, timing of specimen sampling for TDM, and antibiotics allergy and penicillin skin testing. Some ID pharmacists are faculty members or part-time faculty members teaching ID pharmacotherapy to pharmacy students. Speak at national ID and non-ID conferences. Providing nationwide continuing education lectures and workshops. Speak publicly on TV, radio, and different social media platforms about public health matters related to ID. Organize or participate in public health campaigns as a community service. Educate healthcare providers including pharmacy staff on antimicrobials, new guidelines, and policies.
Research/Scholar	• Conduct and publish clinical studies. Supervise pharmacists, pharmacy residents, and students in their ID research projects. Collaborate with physicians in prospective clinical trials and writing of case reports. Contribute to the literature by writing review articles and perspectives.

ASP, antimicrobial stewardship program; DDD, defined daily dose; DOT, days of therapy; HIV, human immunodeficiency virus; ID, infectious diseases; IV, intravenous; PGY, postgraduate year; PO, oral; KSA, Kingdom of Saudi Arabia; PK/PD, pharmacokinetic/pharmacodynamic, TDM, therapeutic drug monitoring.

## 5 Antimicrobial stewardship programs in KSA

Antibiotics are a breakthrough medical discovery in the early 20th century that transformed the health and well-being of humans by reducing the morbidity and mortality of ID (Barlam et al., 2016). Currently, antibiotics are facing threats due to the emergence of AMR and the slow development of new antibiotics. These threats warrant the implementation of ASPs across a wide spectrum of healthcare settings to optimize the utilization of the existing and newly developed antibiotics, hinder the emergence of AMR, and improve economic outcomes. Infectious diseases pharmacists are key members of ASPs as they are regarded as the “Pharmacy Experts” according to the Core Elements of Hospital ASP document by the Centers for Diseases Control and Prevention ([CDC] CDC Stewardship Core Element). In 2010, a statement by the ASHP was published about the role of pharmacists in the ASP and infection prevention (ASHP Pharmacist’s Role in Antimicrobial Stewardship). The Infectious Diseases Society of America in collaboration with the Society of Infectious Diseases Pharmacists and other ID societies recommend the implementation of ASPs to meet these goals.

A national survey by Alghamdi et al. evaluated ASP implementation and factors associated with it in MOH hospitals in KSA (Alghamdi et al., 2021). The survey was sent to 274 MOH hospitals and only 147 hospitals responded indicating a response rate of 53.6 %. The survey revealed that only 39 (26 %) MOH hospitals implemented ASP. Factors related to the low implementation rate include low perception of benefits of ASP, lack of resources, difficulty in the implementation, and high workload. Moreover, 85 % of the responders reported that they are not ready to implement ASP at their organizations due to a lack of knowledge and technological resources. This study was limited only to MOH hospitals and did not include hospitals from other governmental or private sectors. Another study by Alghamdi et al. evaluated the barriers to implementing ASP in KSA using semi-structured interviews (Alghamdi et al., 2019). The authors found that poor enforcement of ASP policies, the disintegration of teams, poor communication among healthcare professionals, lack of manpower, weak education and training in ASP, and lack of technological resources are among the major barriers to ASP establishment. It is important to highlight that the implementation of ASP in KSA saves healthcare costs as demonstrated by several studies. Al-omari et al. showed that the antimicrobial costs decreased by 28.45 % in the first year of the ASP implementation (Al-Omari et al., 2020). Moreover, Amer et al. found that implementing ASP in the medical intensive care unit was associated with a reduction in the total cost of antibacterial agents by 59.2 %. A study by Alawi et al. (Alawi and Darwesh 2016) revealed that implementing ASP could save up to 1,225,577 Saudi Riyals per month.

Outpatient Parenteral Antimicrobial Therapy (OPAT) has been established in some hospitals in KSA (Baharoon et al., 2011, Al Shareef et al., 2022). Some studies evaluated the utility of OPAT in a variety of ID such as osteomyelitis, infective endocarditis, pneumonia, bacteremia, and urinary tract infections. These studies have concluded that OPAT was associated with improved clinical outcomes, decreased length of hospitalization, and cost savings.

Implementation of automation and using clinical decision support (CDS) is crucial to supporting the ASP practice (Agrebi and Larbi, 2020, Peiffer-Smadja et al., 2020). There are various applications for CDS that can be utilized in ID pharmacy practice and ASP that include identification of bacterial resistance, rapid diagnostics, aid antimicrobial selection, monitoring antibiotic consumption, shortening time to optimal therapy, identification of *Clostridioides difficile* infections, and prediction of treatment response (Rittmann and Stevens 2019). When paired with rapid diagnostics, CDS can become a powerful ASP tool.

Overall, the implementation of ASP in KSA remains low. The biggest barrier to ASP implementation is the limited number of ID-trained pharmacists in KSA, especially in non-major cities and rural areas. Efforts are needed to increase the number of ID-trained pharmacists and to enforce ASP implementation across various healthcare settings. Several institutions in KSA have implemented OPAT programs with positive findings. Lastly, more efforts are needed to expand the implementation of CDS and automation to aid increase ASP implementation.

## 6 Infectious diseases pharmacy education and training in KSA

With the evolution and advancement of clinical pharmacy in KSA, several areas of clinical pharmacy have developed in pharmacy education including ID. During the pharmacy education (Bachelor of Science degree in pharmacy or PharmD), students study microbiology and ID pharmacotherapy in didactic lectures and apply it during the internship year (i.e., advanced pharmacy practice experience [APPE]) of the PharmD program. It is important to highlight that students undergoing ID APPE may obtain the maximum educational benefit when they are trained by clinical pharmacists specialized in ID for broad coverage in ID pharmacotherapy. During the general pharmacy practice residency, ID rotation is a core rotation required for completion of the residency program. More specialized training can be achieved by pursuing a clinical pharmacy residency in the ID pharmacy (*R*3).

Only one study investigated the ID curricular content in pharmacy schools in KSA (Alqahtani et al., 2021). This cross-sectional questionnaire was sent to 26 pharmacy schools and aimed to assess the ID subjects covered in the curriculum, teaching strategies, and challenges faced by faculty members in teaching the ID module. This study used the 2019 American College of Clinical Pharmacy pharmacotherapy didactic curriculum toolkit to evaluate ID topics covered in each pharmacy school (Flannery et al., 2020). The toolkit classifies the topics into 3 tiers based on their level of importance (tiers 1, 2, and 3). Twenty-one (80 %) pharmacy schools submitted the completed questionnaire. More than 78 % of respondents indicated that all tier 1 topics are covered in their curricula. Among tier 1 topics, *C. difficile* infection, lower respiratory tract infections, and fungal infections. Among tier 2 topics, most schools (95 %) teach *Mycobacterium tuberculosis* while only 35 % of the schools teach travel medicine. Travel medicine is a crucial topic to teach due to pilgrimage in KSA as people across the globe travel to KSA during Hajj season which could increase the risk of the spread of ID. Miscellaneous viral and bacterial ID in tier 3 topics are covered by most schools as well. Time dedicated to each ID topic varied between schools but mostly ranged between 1 and 3 h per topic. Lastly, fungal infections are a required topic in 86 % of pharmacy schools.

Pharmacy schools in KSA use multiple teaching strategies (Alqahtani et al., 2021). The most consistently used strategies are lectures and practical sessions with facilitated case discussions. More than half of the schools constantly used patient case applications and around 45 % constantly applied an active learning strategy, which is higher than what was previously reported in the US (Jeffres et al., 2019). The use of other teaching strategies, such as student-generated questions, simulations, and concept maps varied across institutions. Also, APPE is essential training for PharmD students during the internship year where they can learn pharmacy practice skills in ID pharmacy.

Regarding postgraduate training, there are 6 ID pharmacy residency programs in 6 training sites offering eight seats in 2022. These programs are accredited by Saudi Commission for Health Specialties (SCFHS), which is the major accrediting body of medical and health sciences residency programs according to published accreditation standards. In the Programs Accreditation Standards Framework approved by SCFHS, there are 5 core rotations and 6 elective rotations in the ID pharmacy residency curriculum. The core rotations include general (non-intensive) ID, intensive care ID, ID in immunocompromised patients (transplant and hematology/oncology), microbiology, and Human Immunodeficiency Virus. The elective rotations include ASP, critical care, hematology/oncology, organ transplantation, infection control, and pediatric ID. Each rotation is 5-week long. The SCFHS has developed standards for each program it accredits, including the ID pharmacy residency program. The standards consist of several sections, including program entry requirements, program rotations and duration, teaching methods, assessment and evaluation, program and course evaluation, description, competencies, goals, and objectives of each rotation, as well as policies and procedures. These standards ultimately seek to guide trainees to become competent and well-rounded in the ID specialty and to match the highest standards as clinical ID pharmacists. Notably, promotion to specialty residency training (*R*3) and graduation from the residency programs requires passing the end-of-year exams, as well as an Objective Structured Clinical Examination and Structured Oral Examination according to SCFHS requirements. The latter 2 tests involve simulation of patient cases and interview-based questions and answers on a case, respectively. The US Board of Pharmacy Specialties (BPS) offers certification in ID pharmacy (BCIDP) through passing an exam (BPS Statistics). This certification should not replace clinical training and residency and can be obtained as continuing post-graduate education. In 2022, there were 1,592 pharmacists who obtained BCIDP around the world. Out of them, 70 obtained it from KSA, making KSA the second country with the highest number of BCIDP obtainers after the US.

## 7 Infectious diseases pharmacy in research

Generally, health-related research performed in various regions in the KSA is required, particularly in the field of ID. Research related to ID is unique compared to other specialties because the type of ID and AMR varies from region to region in KSA. To elaborate, ID pharmacists can publish research projects on topics related to ID pharmacotherapy and ASP projects that evaluate and document the value and the roles of ID pharmacists in various settings (Goff et al., 2020). Although ID pharmacists are actively involved in observational studies, prospective interventional studies should not be overlooked. Notably, ID pharmacists are involved in clinical practice guidelines development given they are pharmacy expertise (Alhazzani et al., 2022). Moreover, ID pharmacists can publish ASP research discussing their experience in establishing these programs and implementing the ASP interventions, as well as evaluating the outcomes of ASPs. Identifying the pharmacists’ roles in ASP and the impact of pharmacist-driven or pharmacy-led ASP interventions is of paramount importance in KSA. Our PubMed search from inception to May 01, 2022, using the terms “antibiotic stewardship” or “antimicrobial stewardship” and both “pharmacy” and “Saudi” with the author affiliation function, identified only 69 papers with the majority of them on inpatient settings. The low number of ASP publications can be explained by the limited number of ID pharmacists in KSA and the ASP implementation rate.

The US has several pharmacy fellowship programs specialized in ID to prepare pharmacists to become independent researchers (Badreldin et al., 2021, Korayem et al., 2021). Pharmacy fellowship programs specialized in ID have not yet been established in KSA. Unfortunately, there is only one pharmacy fellowship program in the Ambulatory Care specialty, which is offered by the College of Pharmacy at King Saud University (KSU) in Riyadh (Fellowship Program in KSU). Nonetheless, KSA has several ID pharmacy residency programs that mainly focus on clinical practice but also require the completion of one research project during the training. There are several research workshops offered by organizations like the Saudi Pharmaceutical Society and the SSCP that can sharpen ID pharmacists' and pharmacy residents' research skills. Moreover, pharmacy residents can present their research projects at the annual meeting organized by SSCP and the research day organized by SCFHS. These events are called Residents Research Day and involve both podium and poster presentations with a judgment panel evaluating the quality of the projects and naming three winners at the end of the day. Such an event enhances competitiveness among pharmacy residents to work hard on their projects.

There are several barriers for ID pharmacists to conduct research in KSA. In fact, many clinicians, including ID pharmacists, focus on clinical practice and patient care while research may not be a priority. This may be different for the clinical pharmacists who are also faculty members, particularly since promotion in academia mainly depends on the research activity. Nonetheless, some governmental hospitals offer financial incentives to clinicians who publish research. Other research barriers, in general, include limited research foundation and skills, lack of protected time for research, heavy clinical and administrative tasks, limited grant opportunities, and difficulty in data acquisition. A challenge for ID-related research is that not all microbiology laboratories report the minimum inhibitory concentration on culture and susceptibility reports. Therefore, research ideas that require such data may not be feasible. Furthermore, many hospitals still have paper-based medical records and medication administration sheets; hence, an accurate list of antimicrobial agents and their time of administration might be difficult to capture. To evaluate the impact of ASP interventions, it is best to compare the study outcomes before and after the intervention implementation in quasi-experimental studies. Unfortunately, some key data cannot be collected retrospectively for the pre-implementation period. For example, if a de-escalation happens, it can be difficult to tell whether it was done by the ASP team, ID consult team, or the primary team. Another barrier is that the clinical efficacy of antimicrobial agents can vary between a region/hospital and to another depending on local AMR rates. In fact, it can change within the same center later due to changes in AMR over time. Also, an ASP intervention in one center resulting in positive outcomes may not be generalizable to other centers. Therefore, conducting multicenter studies is crucial to improve the generalizability of the results.

Notably, the creation of the Saudi National Institute of Health (NIH) and the Research Development and Innovation Authority is expected to have a significant positive impact on research in the KSA that include all areas of healthcare research including ID pharmacy research (Saudi NIH, 2022, Research Development and Innovation Authority, 2022). These research agencies will focus on determining research priorities in KSA, streamlining the policies and regulations to support researchers in KSA across different sectors, creating lectures and workshops to advance research skills, supporting innovation in research, embracing collaboration across different institutions in KSA, and help researchers to find a funding source for their research. Therefore, the Saudi NIH and the Research Development and Innovation Authority can transform the future of research in the KSA.

## 8 Future directions

There is remarkable progress in clinical pharmacy in KSA overall and ID in particular, including pharmacy practice, pharmacy education, and research. Clinical pharmacists play pivotal roles with multidisciplinary teams to provide optimal patient care with personalized and cost-effective recommendations in different areas of clinical pharmacy including ID. However, there are areas that need more efforts for development. Antimicrobial resistance remains problematic in KSA which can be attributed to several reasons, mainly inadequate ASP implementation in KSA. Leaders and policymakers should focus on stronger efforts to implement ASP and the development of local ID treatment guidelines. Moreover, not all the hospitals in KSA have electronic health record (EHR) systems for automation and CDS for efficient implementation of ASP. Leaders in the field especially MOH administrators should work towards higher implementation of automation and CDS to improve ASP implementation in KSA overall. Although clinical pharmacy achieved major advances in KSA, the number of ID-trained pharmacists remains low, particularly in non-major cities. Efforts are needed to increase the number of ID-trained pharmacists through more scholarships to the US, establish more residency seats (especially in non-major cities), and increase the collaboration between major cities and non-major cities. In return, these efforts will improve ASP implementation and other aspects of ID pharmacy in KSA. Research on the scope of clinical pharmacy and ID particularly is growing in KSA. Pharmacy organizations such as SSCP and SPS should encourage pharmacists in the ID and other fields on the positive impact of conducting research through promoting more research activities and organizing research training workshops. A complete list of the current challenges and potential recommendations is available in Table 2.

**Table 2:** Challenges and recommendations for ID pharmacy in KSA.

**Challenges/Areas for Improvement**	**Potential Recommendations**
Many ASPs are managed by physicians and not all hospitals have systems to involve ID pharmacists in ASP activities. In other institutions, ASP are managed by ID pharmacists only and physicians are not involved.	Increase the number of ID pharmacists and physicians trained on ASP and facilitate their collaboration for best clinical outcomes.
The ASP systems in most hospitals are paper-based system and lack automation and clinical decision support.	Invest in ASP electronic health records platform.
ASP in some hospitals is conducted by pharmacists are not specialized ID (i.e., did not complete ID pharmacy residency). The background of such pharmacists may vary, and they may not have solid understanding of and clinical experience in microbiology, ID, and pharmacology of antimicrobial drugs.	Increase the number of ID trained pharmacists and the number of residency program seats in KSA.
Some hospitals in KSA do not have a structure and job description for the ID/ASP pharmacist.	Develop ID/ASP pharmacist work structure and job description.
Not all hospitals have ID physicians, which may limit ID pharmacists to function in full capacity.	Increase the number of physicians trained in ID.
Not all hospitals have antimicrobial committee.	Encourage hospitals to establish antimicrobial committee.
Requirements for clinical pharmacy faculty members vary between the universities in KSA with some universities approve local residency training while others only allow the completion of such training in the US. It is important that such universities be aware that the opportunities in the US are competitive and limited, which may end up with the candidate having to obtain a Doctor of Philosophy degree in a non-clinical specialty.	Increase awareness about tracks for clinical pharmacy education for policymakers in KSA and encourage more efforts to facilitate Saudi scholars to obtain residency training.
Lack of ID pharmacy fellowship in KSA.	Encourage university and hospitals in KSA to establish ID pharmacy fellowships.
In some Saudi universities, ID clinical pharmacists are separated from clinical practice to focus on academic tasks like teaching, research, and administration.	Encourage more integration between practice and academic sites via discussion with policymakers.
Some ID pharmacy residency training sites do not expose the resident to all the elements of ASP.	Encourage the implementation of all ASP elements as in IDSA and CDC ASP guidelines, as well as ID pharmacy residency structure in ASHP residency manual.
Some hospitals hire PharmD graduates without obtaining residency training to work as ID clinical pharmacists.	Encourage more trained pharmacists specializing in ID to establish programs and have the correct route to practice as an ID pharmacist.
Many pharmacists indicated they can work easily with ID physicians in the US and Canada. This might be because those physicians were used to work with ID pharmacists there.	Increase awareness of appropriate ASP and ID practice structures adopted from the North American system.
There is a gap for ID pharmacists to conduct PK/PD research due to lack of specialized laboratories.	Encourage the systems and policymakers to establish PK/PD laboratories for ID pharmacists and pharmacologists to have more research activities and secure funding from Saudi research agencies.
Some institutions hire only one ID pharmacist to cover the clinical service, education and training, and scholarly activities which could lead to burnout and suboptimal coverage.	Encourage institutions to hire more than one ID pharmacist to cover the all the services and tasks to ensure optimal coverage.
Non-major cities lack specialized ID trained pharmacists or have very limited number.	More efforts are needed to increase the number of ID specialized pharmacists in non-major cities.
The number of cost-effectiveness studies on the ASP is limited.	Encourage researchers and healthcare agencies to conduct more cost-effectiveness studies to highlight the positive financial impact of implementing ASP programs.
The number of ID research projects related to ASP is limited especially in outpatient settings.	Increase ID pharmacists’ involvement in ASP research projects in variety of settings including inpatient and outpatient.
Pharmacy students undergoing ID APPE may not be trained by an ID clinical pharmacist.	Encourage pharmacy colleges and hospitals in KSA to train pharmacy students completing ID APPE by an ID clinical pharmacist.
ID pharmacists’ involvement in research projects and publications is limited outside the academic fields.	Encourage ID pharmacists to publish more research projects related to ID pharmacotherapy and projects evaluating the value and the roles of ID pharmacists in various settings.
There is a low involvement of ID pharmacists in the development of clinical practice guidelines in KSA.	Encourage ID pharmacists’ participation in the development of clinical practice guidelines given their pharmacy expertise.
There is a limited participation of ID pharmacists in white papers, position papers, and clinical consensus statements.	Encourage ID pharmacists to be more active in publishing white papers, position papers, and clinical consensus statements.

ASHP, American Society of Health-system Pharmacists; ASP, antimicrobial stewardship program; CDC, Centers for Disease Control and Prevention; ID, infectious diseases; IDSA, Infectious Diseases Society of America; OPAT, outpatient parenteral antimicrobial therapy; PGY2, postgraduate year 2; PharmD, Doctor of Pharmacy; PK/PD, pharmacokinetic/pharmacodynamic; KSA, Kingdom of Saudi Arabia; US, United States.

## 9 Conclusion

Infectious diseases pharmacy has sustained remarkable progress in KSA over the past 3 decades in several areas, including pharmacy practice, education, and research. Additional efforts are needed for further development in ID pharmacy in KSA. Specifically, expanding the use of CDS in EHRs, developing national and institutional clinical practice guidelines, and increasing ASP implementation. These efforts will promote prudent use of antimicrobials and halt the emergence of AMR. The biggest barrier to achieving these goals is the inadequate number of ID-trained pharmacists, especially in non-major cities. Thus, increasing the number of ID-trained pharmacists is a key to achieving the KSA Vision, 2030, KSA Vision, 2030 for better healthcare.

## Declaration of Competing Interest

The authors declare that they have no known competing financial interests or personal relationships that could have appeared to influence the work reported in this paper.
